# Gas sorption porosimetry for the evaluation of hard carbons as anodes for Li- and Na-ion batteries

**DOI:** 10.3762/bjnano.11.106

**Published:** 2020-08-14

**Authors:** Yuko Matsukawa, Fabian Linsenmann, Maximilian Arthur Plass, George Hasegawa, Katsuro Hayashi, Tim-Patrick Fellinger

**Affiliations:** 1 Department of Applied Chemistry, Graduate School of Engineering, Kyushu University, 744 Motooka, Nishi-ku, Fukuoka 819-0395, Japan; 2 Chair for Technical Electrochemistry, Department of Chemistry, Technical University of Munich, Lichtenbergstr. 4, Garching bei München 85748, Germany; 3 Nanochemistry Department, Max Planck Institute for Solid State Research, Heisenbergstraße 1, Stuttgart 70569, Germany; 4 Institute of Materials and Systems for Sustainability, Nagoya University, Furo-cho, Chikusa-ku, Nagoya 464-8601, Japan

**Keywords:** alkaline-ion secondary battery, gas sorption porosimetry, hard carbon, irreversible capacity, ultramicroporosity

## Abstract

Hard carbons are promising candidates for high-capacity anode materials in alkali metal-ion batteries, such as lithium- and sodium-ion batteries. High reversible capacities are often coming along with high irreversible capacity losses during the first cycles, limiting commercial viability. The trade-off to maximize the reversible capacities and simultaneously minimizing irreversible losses can be achieved by tuning the exact architecture of the subnanometric pore system inside the carbon particles. Since the characterization of small pores is nontrivial, we herein employ Kr, N_2_ and CO_2_ gas sorption porosimetry, as well as H_2_O vapor sorption porosimetry, to investigate eight hard carbons. Electrochemical lithium as well as sodium storage tests are compared to the obtained apparent surface areas and pore volumes. H_2_O, and more importantly CO_2_, sorption porosimetry turned out to be the preferred methods to evaluate the likelihood for excessive irreversible capacities. The methods are also useful to select the relatively most promising active materials within chemically similar materials. A quantitative relation of porosity descriptors to the obtained capacities remains a scientific challenge.

## Introduction

Lithium-ion battery (LIB)-based energy storage devices have been gaining high interest in the recent years in many industrial branches, ranging from electronic devices over battery electric vehicles (BEVs) to applications in grid energy storage. Since for grid energy storage a large amount of installed absolute capacity (rather than specific capacity) is required and LIB cells are still expensive, sodium-ion batteries (SIBs) become interesting [1–2]. Compared to Co, which is still an essential component for state-of-the-art LIB cathode materials, Li is much more abundant in the earth’s crust [3–4]. Nevertheless, the market for mobile applications is increasing, especially for new BEVs, and thus the availability of Li and its price will most likely become an issue [5]. Accordingly, for grid energy storage the priority is shifting from energy density to production cost and longevity [6]. Since Na is the fourth most abundant element in the earth’s crust, the production cost for typical precursors is by one order of magnitude less expensive [7]. LIBs and SIBs have different applications. While LIBs are used as traction batteries in BEVs, in which volumetric energy density is crucial for a long mobility range, SIBs are mainly used for grid energy storage applications due to their lower cost [8].

Historically, the first LIB introduced by Sony Corp. used a slightly disordered carbon, a so-called soft carbon (SC), which is graphitizable at temperatures of ca. 3000 °C, and later, from 1992, a more disordered hard carbon (HC), which is not graphitizable at temperatures of ca. 3000 °C as negative electrode [9]. We herein refer to HCs as strongly disordered carbons (having a high fraction of sp^3^-hybridized defects or heteroatoms), independent of their graphitizability. After the introduction of the LIB, efforts in research and development on sodium-ion anodes, i.e., lithium-analogue materials, was the next logical step. Among the alkaline metal ions Na^+^ is the second lightest and smallest ion and still possesses a relatively low standard reduction potential of −2.7 V vs SHE (compared to −3.04 V vs SHE for Li), which is crucial to allow for high energy densities [10]. Many studies were carried out to understand the storage mechanisms of both lithium and sodium ions inside many different carbons. Due to the progress in LIB research and the implementation of stoichiometric and highly reversible graphite anodes (forming LiC_6_), disordered carbons were considered less. Although the volume expansion of HCs during lithium intercalation is lower than that of graphite, implying longer lifetimes, the relatively lower volumetric energy density due to their lower density and lower energy efficiency were detrimental for their commercial usage in the uprising market of portable devices. In 1997, the market share of HC and graphite was still 52% and 43%, respectively, and today graphite is almost exclusively used as negative electrode material in commercial LIBs [11]. Graphite with an interlayer distance of 0.335 nm cannot be intercalated by sodium without solvent co-intercalation [9,12–13]. Theoretical studies suggest a minimum distance of around 0.37 nm in order to enable reversible intercalation of sodium ions [14]. Therefore, the use of less crystalline carbon electrodes in SIBs cannot be avoided [15]. The lower gravimetric energy density compared to LIBs (because of the higher specific weight of Na), the lower volumetric energy density (because of the need for the less dense disordered carbons), as well as the more complicated storage mechanism complicate the commercialization SIBs.

With the current strong interest in electromotive driving and the transformation of the energy grid to sustainable and decentral electricity generation and storage, the unconventional LIB and SIB technology using disordered carbons is currently having a comeback with a strongly increasing number of recent publications (Supporting Information File 1, Figure S1). Conventional LIB technology based on graphite only allows for a limited mobility range, therefore, HCs are investigated regarding the potential for higher energy densities. The seasonal and daytime-dependent character of solar and wind energy requires stationary electricity storage for times of light and wind shortage, with a focus on low cost.

To elucidate the more complex storage mechanism of both lithium and sodium ions in disordered carbonaceous materials, much effort has been spent and many different theories were debated, which are still under discussion [16–18]. It was found that the achievable amount of sodium that could be inserted into carbon depends on the type of carbon. Both lithium and sodium are suggested to have two consecutive but very different charging mechanisms [17,19]. Characteristic charge–discharge curves of both SIBs and LIBs feature two regions, first, a sloping region having a hysteresis between charge and discharge, and second, a plateau appearing at low voltage. The regions may be assigned to the insertion of alkali ions between differently stacked carbon sheets in the sloping region on the one hand. On the other hand, alkali ion adsorption within nanopores in a “plating-like” adsorption process may explain the low voltage plateau due to multilayer adsorption. A direct correlation between the specific cumulative volume of pores smaller than 0.7 nm (ultramicropores) with the respective sodium storage capacity in cellulose-derived carbon showed decreasing sloping capacity and increasing plateau-like capacity with decreasing ultramicroporosity [20]. This finding is in contrast to the results from Dahn and co-workers [17,19]. Apparently, the understanding of the role of specific textural features of disordered carbons for alkaline ion storage remains difficult, also leaving uncertainty about the potential of carbon materials for commercial application.

Besides the remaining questions about the reversible capacity, the reduction of the irreversible capacity for disordered carbons is another important field of research crucial for commercialization. As already mentioned above, one of the reasons why HCs were gradually substituted by graphite in commercial LIB cells, and one of the main limitations in current SIB research, is the relatively high irreversible capacity due to the formation of a solid electrolyte interface (SEI) layer. The irreversible capacity is believed to originate from electrolyte decomposition at potentials below the stability window of the electrolyte (for LIBs typically around 0.8 V_Li_) [21]. Since the dielectric SEI passivates the electrode, an irreversible capacity proportional to the electrochemical active surface area is expected. Accordingly, the reduction of the specific surface area (SSA) of the anode materials as well as the deposition of amorphous carbon films were shown to reduce irreversible capacity losses [22–23]. Ji et al. found that lower total pore volumes (determined by N_2_ sorption) gave rise to increased reversible sodium storage capacities for sucrose-derived HCs [24]. In contradiction to this study, Yang et al. showed that the pore volume of pores between 0.3 and 0.5 nm is responsible for reversible sodium storage [25]. Yang et al. conclusively argued that ion-sieving effects, determined by a critical pore diameter, differentiate between pores contributing to either the reversible or the irreversible capacity [25]. Pores that can be accessed by solvent molecules of the electrolyte will contribute to the irreversible capacity, while smaller ones are suitable for the adsorption of alkali metal ions protected from side reactions with solvent molecules from the electrolyte. It was further shown that additional irreversible capacity can arise from alkali metal ions reacting with surface defects or reactive surface groups and small molecules other than the electrolyte adsorbed to the walls of nanometric pores that were not removed during the cell production [17]. These unwanted side reactions are expected to reduce the reversible capacity that can be obtained by adsorption within the pore system.

In this work, we use different gas sorption porosimetry (GSP) techniques to investigate surface areas and porosities, contributed by pores of different size, of different HCs. We critically evaluate these methods for the quantification of parameters related to the reversible and irreversible capacity of the materials as LIB and SIB anodes.

## Results and Discussion

### Strategies for improving the capacity of hard carbons and revealing the pore structures

At the electrode–electrolyte interphase, HCs can act as molecular sieves. Here, Na ions are adsorbed in the pores in a metal-plating-like mechanism and separated from bigger solvent molecules that suffer from electrochemical reductive decomposition at potentials below ca. 0.8 V_Li_ or towards the alkali metal, respectively. Since the storage mechanisms of lithium and sodium in HC anodes seem to be analogous, this is also expected to be a central effect in LIBs. We consider the determination of the following parameters crucial for the development of improved HCs towards reversible high capacity anodes for next generation LIBs and SIBs: 1) the surface area of the electrode exposed to electrolyte molecules, 2) the critical size of the effectively sieving pore, separating the pore system from the electrolyte, and 3) the quantity of the pore volume inaccessible to the electrolyte, likely being related to the reversible capacity.

The most common method to determine SSAs and nanoporosity in the battery community are nitrogen (N_2_) sorption porosimetry, as well as krypton (Kr) sorption porosimetry in case of very low surface areas. A precise quantification of microporosity (pore diameters smaller than 2 nm), however, cannot be unambiguously achieved by N_2_ sorption alone and not at all using Kr sorption. The quadrupole moment of N_2_ may lead to stronger interactions with carbon features resulting in smaller apparent pore diameters compared to Ar sorption porosimetry [26].

Furthermore, the pore size determination and the quantification of the pore volume of pores as small as the herein relevant (smaller than approx. 0.7 nm) are complicated by the limited accessibility of these pores by N_2_ sorption porosimetry measurements at 77.4 K, due to low kinetic energy – likewise to the electrolyte molecules [26]. The lower vapor pressures of CO_2_ and H_2_O compared to N_2_ allow for measurements at higher temperatures with the respective higher kinetic energies. Because of higher measurement temperatures, CO_2_ GSP at 273.15 K (0 °C) and water vapor sorption at 293.15 K (20 °C) are hence suggested, despite the ambiguous character of the pore size assignments [27]. Considering the expected complex interdependencies of the porosity/pore size distribution of HCs with their electrochemical properties, a combination of these sorption techniques seems reasonable.

To minimize capacity losses due to SEI formation in graphite-based LIBs, the external surface area of electrode materials is commonly minimized (i.e., small particle sizes are avoided). To improve the Brunauer–Emmett–Teller (BET) surface area determination, Kr sorption is commonly used. However, Kr sorption (performed at 77.4 K) cannot account for the apparent surface area originating from adsorption inside micropores.

Herein, two different types of HCs prepared with different procedures are compared regarding their morphological characteristics as well as their electrochemical properties. Six samples were prepared via hydrothermal carbonization (HT) followed by pyrolytic carbonization (HT carbons), and two samples were obtained from carbonized resorcinol–formaldehyde resins (RF carbons). The electrochemical sodium storage characteristics of the RF carbons were previously reported [28–29].

### Characterization of hard carbons

**Figure 1:**
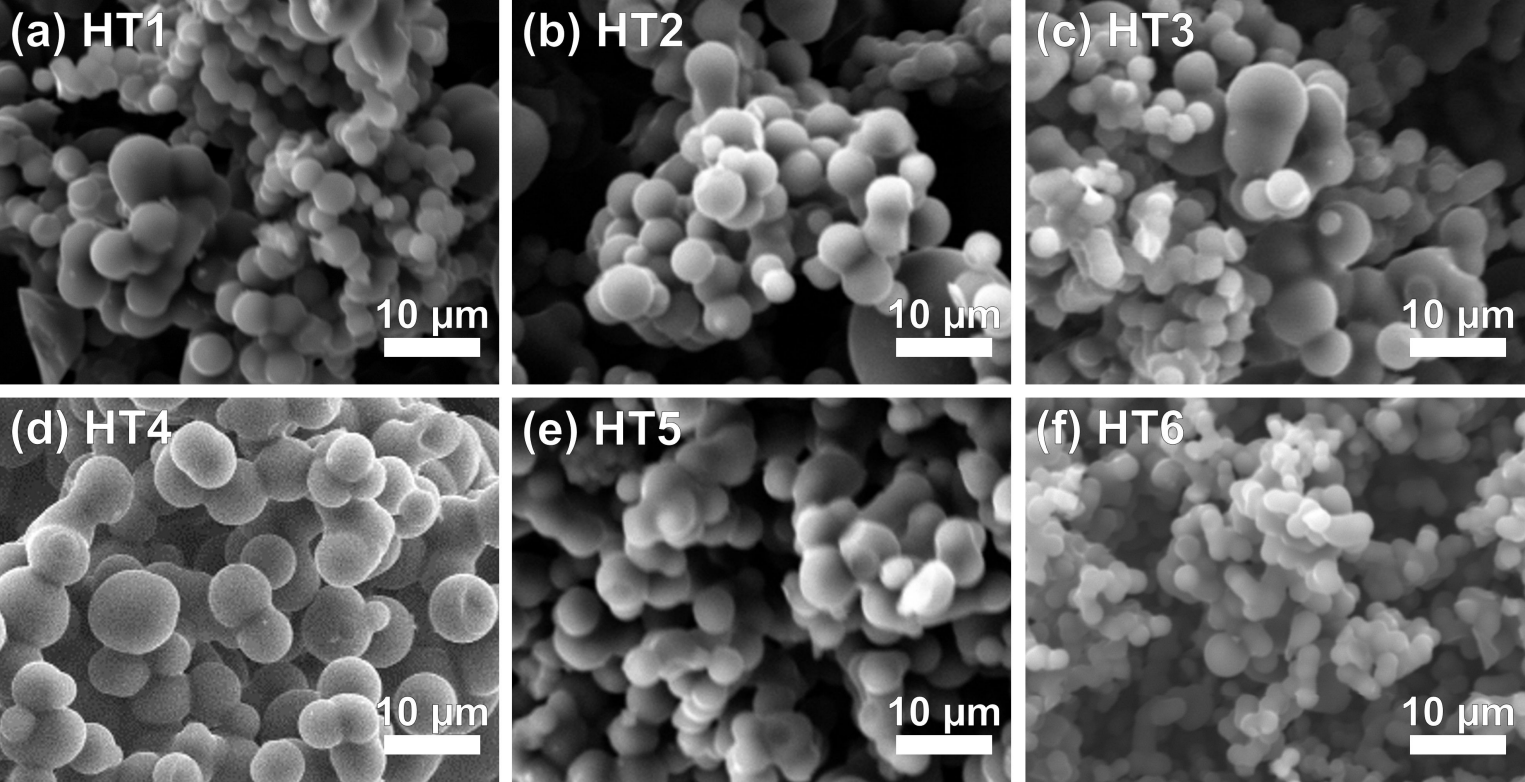
SEM images of HT carbons: a) HT1, b) HT2, c) HT3, d) HT4, e) HT5 and f) HT6.

The X-ray diffraction (XRD) patterns consistently point to disordered, amorphous HCs with missing sharp (002) and (101) reflections (Supporting Information File 1, Figure S2). The described features were also found for the RF samples as reported earlier by Hasegawa and co-workers (Supporting Information File 1, Figure S3) [28–29]. The position of the (002) reflection of the HTs corresponds to large interlayer distances in the range of 0.4 nm, while the RF carbons show *d*-spacings of 0.43 and 0.39 nm. Raman spectra of HT2 shows the G band peak at ca. 1590 cm^−1^ and in addition the D band peak at ca. 1340 cm^−1^, which is further pointing to a disordered, defective graphitic structure (Supporting Information File 1, Figure S4). The D-to-G intensity ratio is 0.98, while it is 1.12 and 1.29 for, respectively, RF-1000 and RF-1600 (Supporting Information File 1, Figure S5), all indicating highly disordered carbons having a large fraction of sp^3^ hybridization. The morphologies of the HT samples are very similar (Figure 1), but show a different morphology compared to the RF carbons (Supporting Information File 1, Figure S6). Scanning electron microscopy (SEM) imaging reveals spherical particles, morphologically similar to previously reported HCs derived from the hydrothermal carbonization of saccharides [30–31]. Despite the slightly different preparation protocols, morphological differences between the hydrothermally obtained HCs are not significant (Figure 1). The two RF carbon samples showed well-defined monolithic macrostructures obtained by spinodal phase separation during the preparation [28–29]. After grinding for preparation of the electrodes, however, the monolithic structure was lost (Supporting Information File 1, Figure S6). The reason why HT6 (Figure 1f) shows smaller particle diameters than the other carbons (Figure 1a–e) can be explained by the secondary catalytic effect of borax during the hydrothermal carbonization of sugars [32].

### Gas sorption porosimetry of hard carbons

**Figure 2:**
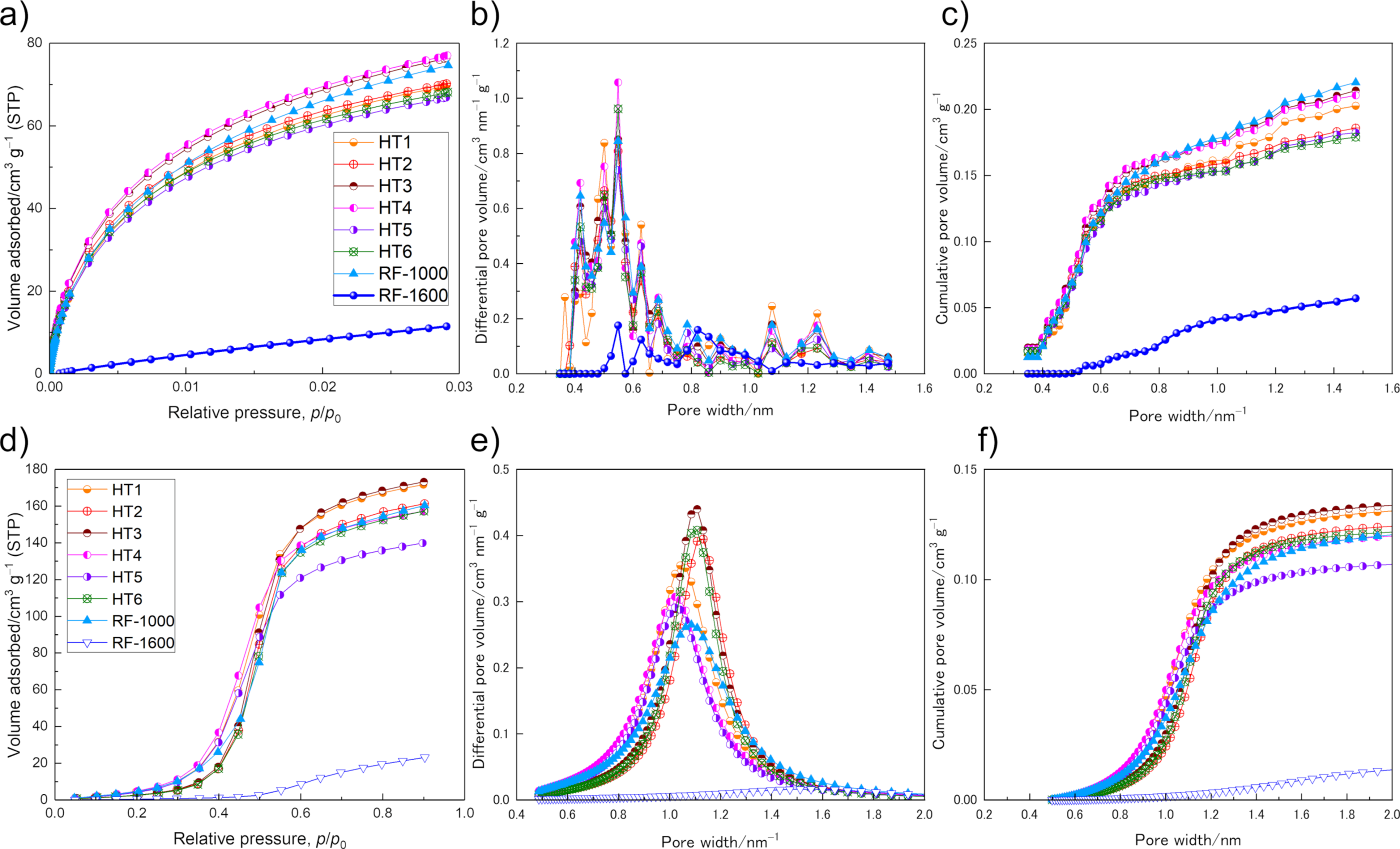
Gas adsorption isotherms (a, d), calculated pore size distributions (b, e), and cumulative pore volumes (c, f) for CO_2_ (a–c) and for H_2_O (d–f) adsorption (STP-standard pressure).

N_2_ sorption porosimetry of the HT samples turned out to be problematic, since the equilibrium conditions were not reached (exemplarily shown in Supporting Information File 1, Figure S7). We expect the combination of large, micrometer-sized pore systems with pore necks of a few angstroms and kinetic limitations of the N_2_ molecules to penetrate the pore system. Kr sorption at 77.4 K as well as CO_2_ and H_2_O sorption measurements at higher temperatures were therefore performed and the resulting isotherms, pore size distributions, and cumulative pore volumes for CO_2_ and H_2_O are shown in Figure 2.

The CO_2_ isotherms for the HT samples show relatively high CO_2_ sorption capacities with similar isotherm shapes (characteristic Langmuir isotherms). The CO_2_ uptake values of the HTs were in the range of 60 to 80 cm^3^ g^−1^ (roughly corresponding to 2.7 to 3.6 mmol g^−1^) reflecting relatively similar porosities (Figure 2a). The isotherm for RF-1000 is also very similar to those of the HT samples; however, for RF-1600 the CO_2_ uptake is much lower (11.5 cm^3^ g^−1^ or approx. 0.51 mmol g^−1^).

As indicated by the high CO_2_ uptakes, the apparent SSAs derived from CO_2_ sorption are also relatively high with a deviation of 14% for the HT samples ranging from 468 m^2^ g^−1^ (HT5) to 544 m^2^ g^−1^ (HT3) (Table 1). The SSAs for RF-1000 and RF-1600 are 546 and 270 m^2^ g^−1^, respectively. Importantly, the obtained values for the SSA from CO_2_ sorption (SSA_CO2_), for all samples, strongly differ from values determined by Kr sorption (SSA_Kr_). Table 1 summarizes the SSAs calculated from CO_2_ and Kr sorption measurements using the BET theory.

**Table 1:** Properties of the carbon samples from Kr, CO_2_ and H_2_O sorption.

sample	SSA_Kr_^a^ (m^2^ g^−1^)	SSA_CO2_^b^/SSA_N2_^c^ (m^2^ g^−1^)	CPV_CO2_^d^/CPV_N2_^e^ (cm^3^ g^−1^)	CPV_H2O_^f^ (cm^3^ g^−1^)

HT1	0.60	492^b^	0.138^d^	0.00584
HT2	1.41	486^b^	0.141^d^	0.00332
HT3	2.66	544^b^	0.152^d^	0.00376
HT4	1.29	535^b^	0.155^d^	0.00633
HT5	0.89	468^b^	0.134^d^	0.00523
HT6	8.11	471^b^	0.138^d^	0.00336
RF-1000	8.56	546^b^/630^c^	0.145^d^/0.188^e^	0.00574
RF-1600	14.69	270^b^/3.8^c^	0.014^d^/0.0002^e^	0.00047

^a,b,c^Specific surface area obtained by the BET method from Kr, CO_2_, and N_2_ isotherms, respectively. ^d,e,f^Cumulative pore volume (CPV) obtained from Monte Carlo, density functional theory, and Mahle method for CO_2_, N_2_ and H_2_O in the pore size range below 0.7 nm, respectively.

The absolute values for the SSAs derived from Kr and CO_2_ sorption differ by two to three orders of magnitude indicating a very different physical meaning of surface depending on the employed method. Since the kinematic diameters of the battery electrolyte molecules are of the same order of magnitude as the gas molecules used (and at the same time large compared to Li and Na ions), a careful examination of the validity of the respective methods is a sine qua non. While the SSA_Kr_ of HT6 (8.11 m^2^ g^−1^) is at least three times larger than those of other HT samples (Table 1), this trend is absent in case of CO_2_ sorption. The results for Kr reflect the observed higher geometrical surface-to-volume ratio of HT6 compared to the other HC samples, as mentioned above (Figure 1). Obviously, Kr adsorbs at the outer (external) surface of the particles, but does not penetrate the small pores of the particles at the measurement temperature of 77.4 K. For the CO_2_ sorption, this outer surface is much smaller and therefore negligible compared to the large internal surface area that is accessible with CO_2_. However, the SSA_Kr_ of HT6 is still smaller than that of both RF carbons, with RF-1600 showing by far the highest SSA_Kr_. From the perspective of graphite anodes, considering the external surface area to be directly related to SEI losses, RF-1600 would be expected to have 2–20 times higher irreversible capacities than the other samples.

In contrast to the HT samples, N_2_ sorption was successfully carried out on the RF samples. Comparison of the values obtained from CO_2_ versus N_2_ shows a 13% lower SSA_CO2_ for RF-1000 (546 m^2^ g^−1^_CO2_ compared to 630 m^2^ g^−1^_N2_), while for RF-1600 the SSA_CO2_ is 71.1 times higher than the SSA_N2_ (270 m^2^ g^−1^_CO2_ compared to approx. 4 m^2^ g^−1^_N2_). Obviously, at 77.4 K small structural differences in the pore systems decide whether or not N_2_ molecules can penetrate.

It is important to highlight that the deviations are not artefacts from the applied models, but are real effects resulting from the unequal accessibility of the small pores for the different gas molecules. In HC anode research the use of CO_2_ sorption rather than N_2_ sorption thus appears to be more reasonable.

Pore size distributions of the HT carbons derived from CO_2_ sorption data using the Monte Carlo method were centered around 0.5 nm with less abundant larger pores between 0.7 and 1.5 nm (Figure 2b). RF-1600 does not have pores smaller than 0.5 nm with main porosity contributions between 0.6 and 1.1 nm. The corresponding cumulative pore volumes of the HT samples as well as of RF-1000 show that roughly two thirds of the porosity are originating from pores smaller than 0.7 nm, the so-called ultramicropores (Figure 2c). Porosity contributions for RF-1600 are more homogeneously distributed, however, at much lower absolute numbers. Despite having the highest geometrical surface-to-volume ratio, RF-1600 is likely to have a much smaller electrode–electrolyte interface than the other samples.

The H_2_O sorption measurements also reveal similar isotherm shapes for the HT samples and for RF-1000, but not for RF-1600. The characteristic sigmoidal shapes for the HT samples, however, show different relative adsorption onset pressures, indicating differences in hydrophilicity or average pore size as well as slightly different slopes for the gas uptake (Figure 2d). Similar to the results of CO_2_ sorption, RF-1600 shows a relatively low uptake. The adsorption branch is furthermore shifted to higher relative pressures compared to the other samples, clearly pointing to a less accessible pore system. According to the Mahle model [33] and the Wang equation [34], the center of the pore diameter distribution of the HT samples is at around 1.0 nm for HT4 and HT5, while it is around 1.1 nm for HT2, HT3, and HT6 (Figure 2e). HT1 and RF-1000 lie in between. The very similar synthetic protocols for the different HT materials make hydrophilicity differences appear unreasonable compared to differences in the pore system. Because of the different preparation procedures, it remains speculative to assign differences in the water sorption results between RF-1000 and HT samples to different pore characteristics only. However, it is to mention that the isotherm characteristics are generally rather similar, like in the case of CO_2_ sorption. The results illustrate the ambiguous assignment of pore sizes for CO_2_ and H_2_O sorption measurements. However, the Monte Carlo method is an advanced model compared to the Mahle model, resulting in higher reliability in reflecting the pore system for CO_2_ sorption measurements compared to H_2_O sorption. Interestingly, the trends revealed by adsorption of the different gases are not identical. The determined porosity trends are RF-1000 ≈ HT3 ≈ HT4 > HT1 ≈ HT2 > HT5 ≈ HT6 ≫ RF-1600 for CO_2_ (Table 1d) and HT3 ≈ HT1 > HT2 ≈ RF-1000 ≈ HT4 ≈ HT6 > HT5 ≫ RF-1600 in case for H_2_O (Table 1f). While for CO_2_ sorption, the different uptakes are assigned to more abundant pores smaller than 0.7 nm (ultramicropores) in case of HT3 and HT4, H_2_O sorption reveals different characteristic pore sizes. Independent of the total porosity HT1, HT4, and HT5 have smaller characteristic pore diameters than the other HT samples. Again, this observation is not an artefact of the pore size calculation, but can also be clearly observed in the isotherms by the shift in the H_2_O uptake to lower relative pressure values for these samples. A possible explanation could relate the steep gas uptake to different sizes of pore necks, reflecting a barrier to penetrate the whole pore system.

Similar to the case of SSA determination by N_2_ and CO_2_ sorption, it is interesting to compare absolute values and relative deviations in the quantification of pore volumes of the RF carbons using N_2_, CO_2,_ and H_2_O sorption. The total pore volume (TPV) is a simple porosity estimation derived from the total gas uptake at maximum relative pressure in a certain GSP measurement. The TPVs of RF-1000 quantified by CO_2_ and H_2_O sorption are 36.0% and 47.8% smaller than those obtained by N_2_ sorption. Apparently, the absolute quantification of porosity is questionable and the most accurate results may depend on how well the gas fits the studied material. Relative differences for similarly prepared materials may be helpful for the evaluation of HCs for application as anode electrodes in LIBs or SIBs, though. For RF-1000, it can be stated that the total porosity is 6.32, 27.40, and 6.79 times larger than that of RF-1600 according to CO_2_, N_2_, and, H_2_O sorption, respectively.

### Reversible/irreversible capacities of hard carbons and the relationship with the surface structures

**Figure 3:**
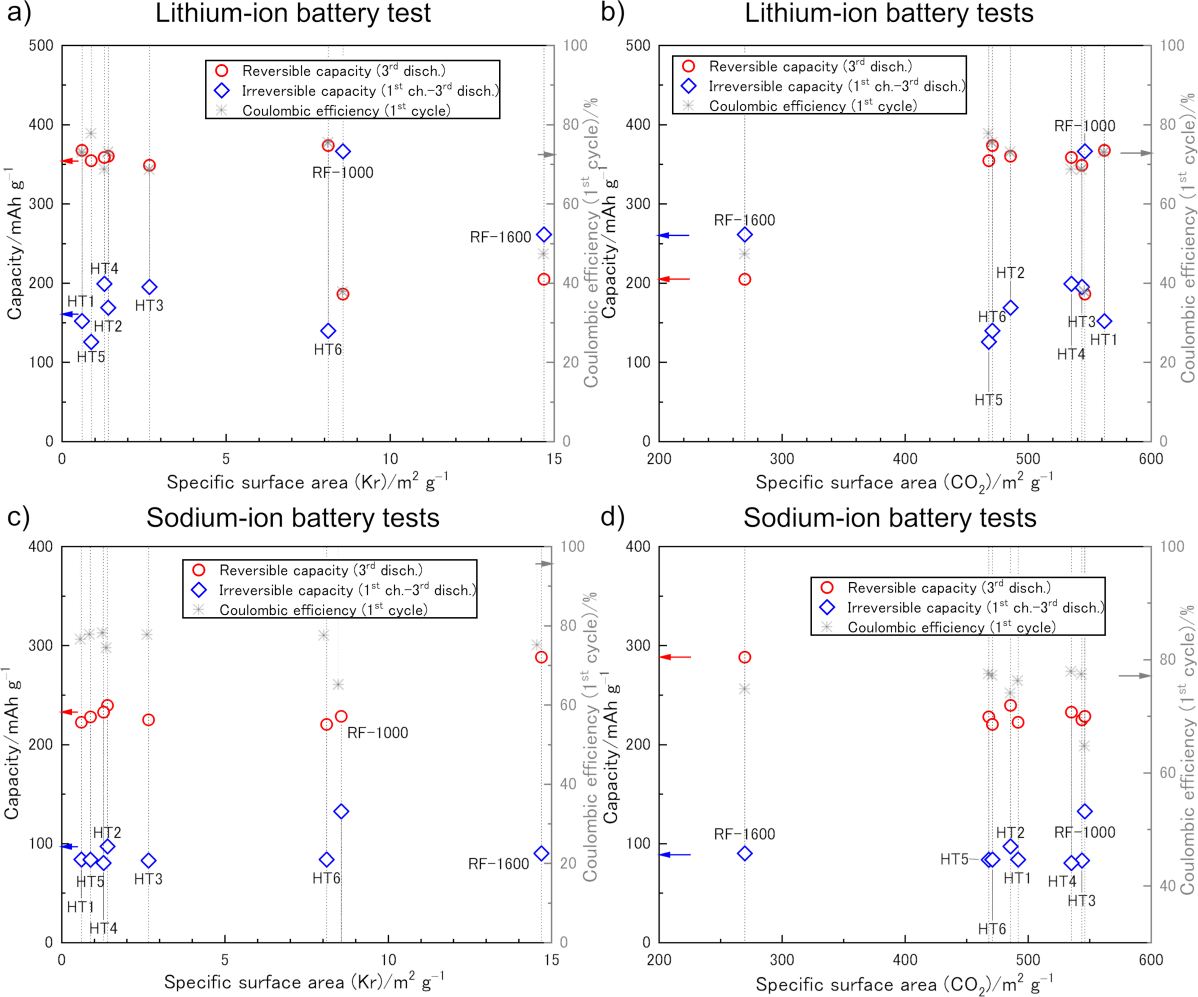
Capacities (reversible: red circle, irreversible: blue diamond, *Y*-axis) and Coulombic efficiencies (gray asterisk, *R*-axis) against SSA obtained from Kr (a, c) and CO_2_ (b,d) adsorption isotherms of lithium (a, b) and sodium (c, d).

**Figure 4:**
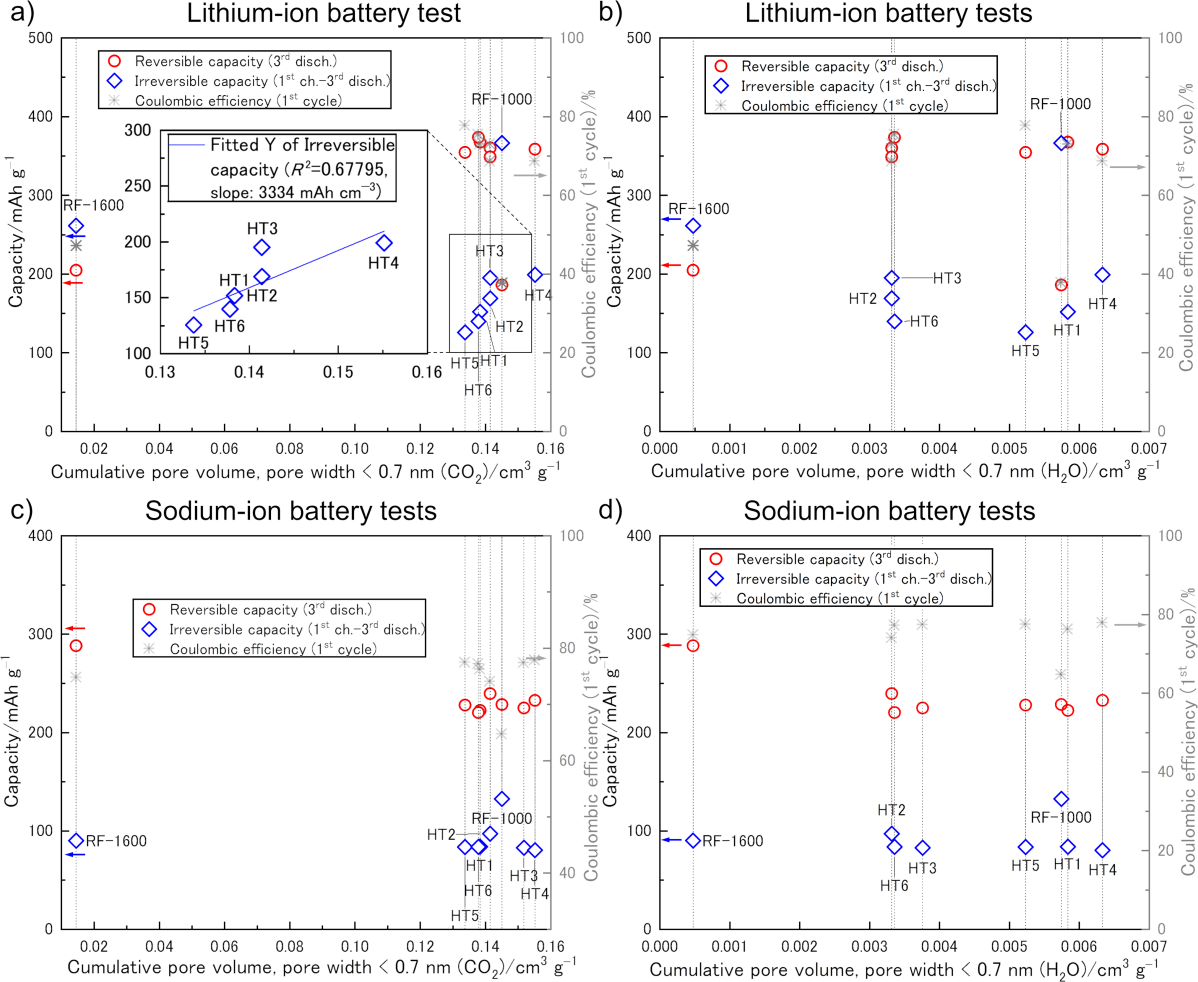
Capacities (reversible: red circle, irreversible: blue diamond, *Y*-axis) and Coulombic efficiencies (gray asterisk, *R*-axis) against cumulative pore volumes (obtained from CO_2_ adsorption by Monte Carlo method (a, c) and from H_2_O adsorption by Mahle method (b, d)) of lithium (a, b) and sodium (c, d). The inset in (a) is an enlargement of the irreversible capacity trend of HT carbons (blue line: approximate straight line of irreversible capacity of HT carbons. Slope: 3334 mAh cm^−3^, coefficient of determination, *R*^2^ = 0.67795).

The mechanistic similarity of gas adsorption experiments to the lithium or sodium discharge process suggests that effects in GSP measurements may be correlated with phenomena in the corresponding battery tests [35]. Electrochemical cycling experiments were carried out in ethylene carbonate (EC)/ethyl methyl carbonate (EMC) = 3:7 (v/v) for LIB tests and in EC/diethyl carbonate (DEC) = 1:1 (v/v) for SIB tests. In both cases, the smaller solvent molecule was EC, which we thought might determine a critical ion-sieving pore size for both LIB and SIB tests. With the aim to relate the investigated morphological features of the HCs to their electrochemical properties as LIB (Figure 3a,b) and SIB (Figure 3c,d) anodes, irreversible capacity and reversible capacity were defined as follows: the irreversible capacity is the difference between the 1st charge and the 3rd discharge capacity. The reversible capacity is defined by the 3rd discharge capacity. The values are obtained from charge–discharge curves, which are plotted as a function of quantities of different morphological features obtained from the GSP measurements (Figure 3 and Figure 4). Since the charge–discharge tests vs lithium were performed in multiple cells for each of the HTs, their capacities and Coulombic efficiencies are average values. We herein regard cumulative pore volumes (CPVs) of pores smaller than 0.7 nm, i.e., pore sizes that could be considered inaccessible to solvent molecules, as well as of pores smaller than 3.0 nm*.* We related the capacities also to other cumulative pore volumes as well as the TPVs in search for direct relations.

The correlation of the SSA_Kr_ with the capacities in LIB (Figure 3a) or SIB (Figure 3c) anodes, as well as with the respective Coulombic efficiencies, revealed no clear trend. In fact, the material with the highest SSA_Kr_ (RF-1600) turned out to have the smallest irreversible capacity, clearly showing that the Kr sorption data are not at all related to the electrode–electrolyte interface in the final battery. HTs with higher SSA_CO2_ tended to show higher irreversible capacities, except HT1, in the case of lithium, it was not seen in the case of sodium (Figure 3b,d). It is to mention that the absolute numbers of the capacity-per-surface-area for the HT samples largely deviate from the RF samples. This indicates that the quantity of the electrochemical reactions, may it be reversible or irreversible, are not only related to the measurable surface area for amorphous electrode materials. One may consider different deposition mechanisms (e.g., van der Merwe layer-by-layer or Volmer–Weber island growth) to explain this. However, it is more likely related to different contributions of porosity that is even inaccessible to CO_2_, i.e., pseudo-graphitic interlayer voids or larger pores that are surrounded by smaller pores.

The same trend was also found for correlations of CPVs of pores smaller than 0.7 nm obtained from CO_2_ sorption (CPV_CO2_) with irreversible capacities, however, not quite for pores smaller than 1.5 nm from H_2_O sorption (CPV_H2O_) (Figure 4). The HT samples with higher CPVs basically showed larger irreversible capacities, again only in the case of LIB tests (Figure 4a,b), while such a trend speculatively remains for SIB tests on the RF carbons, although only based on two samples. Differences between the trends for the irreversible capacities of lithium (Figure 4a,b) and sodium (Figure 4c,d) for HT samples, may be explained with a better utilization of the HT carbons by lithium, as will be further discussed below. The fact that increased irreversible capacities relate to both increasing SSA_CO2_ (Figure 3b) and increasing CPV_CO2_ (Figure 4a,b) points to the fractal nature of HCs, meaning that at such small pore sizes a strict definition of surface area cannot be applied, as reflected in the preferred use of the term “apparent surface area” [36–37]. Another interesting observation is that the pore volumes obtained by CO_2_ and H_2_O sorption, both show no direct relation to the reversible capacity although apparent pore sizes down to approx. 0.35 nm were quantified. This experimental result, in principle, contradicts both Yang et al. [25] as well as Ji et al. [24], since neither the porosity of small pores explains the capacity, nor is a trend of increasing reversible capacity with decreasing pore volumes observed. We rather find that measurable porosity in micrometer-sized hard carbons may cause irreversible capacity (especially in LIBs) and that the characterization of this porosity should rather be carried out with CO_2_ or H_2_O sorption instead of N_2_ or Kr sorption.

Of course, from the results of two RF carbons a trend cannot be concluded. However, it stands out that the slopes of assumed trends for pore descriptors derived from CO_2_ sorption (Figure 3b and Figure 4a) match the ones for the HT carbons relatively well (3334 mAh cm^−3^ for the HC carbons according to the linear fit). This indicates that CO_2_ sorption is indeed the preferred method over N_2_ sorption to estimate irreversible capacity losses by means of SEI formation. Relatively more promising samples may be selected based on this technique, which potentially even allows for the quantification of certain HC anodes. For the RF carbons, at least the ratios RF-1000/RF-1600 of SSA_CO2_, CPV_CO2_, and CPV_H2O_ correspond well to the irreversible capacity ratio, all having a value of ca. 6. This suggests that in case of the RF samples, the penetration of both H_2_O and CO_2_ into the pore system is similar and therefore can be used to mimic the penetration of electrolyte molecules. Furthermore, an obvious difference between LIB and SIB tests is observed for the HT samples. While linear trends are obtained for LIB tests, the SIB tests seem to have no correlation between the irreversible capacity and any porosity descriptor. Thus, it is assumed that the measured porosity of HT carbon particles cannot be fully penetrated by the solvent molecules in SIB tests, potentially because of clogging caused by the larger DEC solvent molecules. Hence, our assumption of EC determining a critical ion-sieving pore size for both LIB and SIB seems to be wrong.

As already mentioned above, the very small pores that are accessible by GSP do not relate to the obtained reversible capacities. This seems to contradict the size range of pores in which sodium can be adsorbed according to Yang et al. [25], since we can fairly well quantify porosities between 0.3 and 0.5 nm. Are these pores not contributing or is it possible that different penetration depths of solvent molecules and, hence, SEI deposits smear out the relation of pore volume to reversible capacity? A simple relation of the porosity that can be filled with sodium or lithium to the corresponding theoretical capacity may be helpful for discussion (*V*^spec^_pore_ is the specific pore volume):

[1] $${}^{\text{Na}}{\text{C}}_{\left[{\text{mAh g}}^{-1}\right]}=1129{\text{ mAh cm}}^{-3}\cdot V^{\text{spec}}{}_{\text{pore}},$$

[2] $${}^{\text{Li}}{\text{C}}_{\left[{\text{mAh g}}^{-1}\right]}=2066{\text{ mAh cm}}^{-3}\cdot V^{\text{spec}}{}_{\text{pore}}.$$

Equation 1 and 2 are based on the simple assumption that sodium and lithium are being plated with their bulk densities on the inside of the pores in the HC anode. When using the CO_2_ sorption data (CPVs) of HT4 (highest porosity) and HT5 (lowest porosity) Equation 1 and Equation 2 reveal expected capacities of, respectively, 175 and 151 mAh g^−1^ for SIB tests and of, respectively, 320 and 277 mAh g^−1^ for LIB tests. Comparison with the third discharge capacities (reversible capacities) shows an underestimation of the capacity of the HT carbons by 25 and 34%, respectively, for SIBs and by 11 and 22%, respectively, for LIBs. As the porosities obtained by H_2_O sorption are consistently smaller than those obtained by CO_2_ sorption the underestimation is even larger with these data.

Since the characteristic sloping as well as the plateau region in the charge–discharge curves are often expected to represent, respectively, intercalation and adsorption, we also approached the correlation of the GSP data with the separated contributions of those two regions. Although, the accessible porosity could now theoretically (according to Equation 1 or Equation 2) explain each of the contributions, a direct relation of the porosity descriptor to either one or the other branch was again not obtained (Supporting Information File 1, Figure S8). Accordingly, it is apparent that the underlying capacity is not related to the measurable porosity. Furthermore, a limited utilization of the carbon anode materials by gas molecules as well as alkaline earth metal ions is assumed. Both will depend on the actual morphology of the hard carbons indicating the complicated reality of HC anodes in SIBs as well as in LIBs.

To improve the understanding of the porosity accessible by GSP techniques more sophisticated techniques are necessary. Especially the connectivity of pores affecting the penetration depth and electrode utilization will play a crucial role in understanding and predicting the sodium and lithium storage capacities of HC anodes, and thereby elucidate the full potential of those materials for LIB and SIB technology.

## Conclusion

We carried out a comparative study of porosity descriptors obtained by different gas sorption porosimetry techniques (Kr, N_2_, CO_2_, and H_2_O) for the relation with the electrochemical performance of HC anodes in lithium and sodium ion battery tests. Different to the case of graphite anodes, the geometry of the particles does not allow for conclusions on capacity losses through SEI formation. The use of Kr sorption to estimate the accessible surface for the used electrolyte solvent molecules by BET surface area measurements leads to a strong underestimation of capacity losses. The surface area values obtained from Kr sorption are two orders of magnitude lower than those obtained from CO_2_ sorption, and can give misleading results regarding expected irreversible losses. In the present study, the sample with the highest relative Kr sorption-based surface area even exhibited the lowest capacity losses amongst the tested samples. The CO_2_ sorption-derived surface area values turned out to be more reliable than the values obtained from the most commonly used N_2_ sorption analysis. N_2_ sorption porosimetry measurements can also dramatically underestimate the pore accessibility for electrolyte molecules and, hence, the SEI formation. Furthermore, the porosity contributed by ultramicropores (0.3–0.7 nm) could not be related directly to the obtained reversible capacities, neither for LIB nor for SIB tests. Instead, it was found that for LIBs even the porosity from pores below 0.7 nm (determined with CO_2_ and/or H_2_O sorption) was proportional to capacity losses due to SEI formation. For SIBs the trends are debatable. However, they point to a limited utilization of ultramicropores of mesoscopic hard carbons in both gas sorption experiments and battery cycling. Finally, it is recommended to employ H_2_O and especially CO_2_ sorption porosimetry for the research on HC anodes to support progress in the understanding of the SEI formation and reversible alkali metal storage.

## Experimental

### Material synthesis

#### Preparation of carbons derived from ᴅ-fructose

The carbons synthesized via hydrothermal method were produced by a step synthesis similar to the reported preparation according to Väli et al. [30] and Fellinger and co-workers [32]*.* The first step was a hydrothermal synthesis of a precursor, followed by a pyrolytic carbonization to get the final HC product. At first, a 25 wt % solution of ᴅ-fructose (Sigma-Aldrich, Germany) in deionized water (Millipore, Merck, Germany) was prepared. Similar to a synthesis reported by Fellinger et al. [32], Na_2_B_4_O_7_·10H_2_O (Borax) (Sigma-Aldrich, Germany) was used as a structure directing agent for the preparation of one sample. The concentration in the ᴅ-fructose solution was 0.06 wt %. 20 g of the solution with or without borax was poured into a 25 mL quartz tube and was heated in a Teflon-lined autoclave for 24 h at different temperatures from 190 to 220 °C. After cooling down to room temperature, the autoclaves were opened and the black precursors were washed with around 2 L of water and then 1 L of ethanol to remove residuals of the polymerization by vacuum filtration. After drying the precursor overnight at 70 °C under vacuum, it was carbonized in a tube furnace with argon flow at a rate of higher than 0.5 L min^−1^ at 1000 °C for 1, 5, or 10 h. The carbons produced by this hydrothermal method (HT carbons) will be called HT*x*, where *x* is an integer. The synthesis conditions (temperature of hydrothermal treatment and carbonization time under argon flow) are listed in Table 2.

**Table 2:** Synthesis conditions for the six HT carbons.

sample	temperature of HT treatment (°C)	carbonization time (h)	addition of borax during HT treatment

HT1	190	5	—
HT2	200	5	—
HT3	220	5	—
HT4	200	1	—
HT5	200	10	—
HT6	200	5	0.06 wt %

#### Preparation of carbons from resorcinol-formaldehyde gels

Porous carbon monoliths were obtained by carbonization of resorcinol–formaldehyde (RF) gels obtained via a sol–gel process as previously reported [28–29]. In short, 2.20 g of resorcinol (Sigma-Aldrich Co.) was added to a mixture of 4.0 mL of 10 mM HCl aq. (Kishida Chemical Co., Ltd.) and 0.80 mL of ethanol (Kishida Chemical Co., Ltd.) to obtain a homogeneous solution. The solution was cooled to 0 °C and then a formaldehyde solution (37 wt % in H_2_O containing 10–15 wt % methanol) (Sigma-Aldrich, Germany) was added under vigorous stirring. After mixing for 5 min, the solution was kept at 40 °C for 24 h for gelation and then aged at 80 °C for 24 h. The gel was washed with ethanol at 60 °C for 4 h three times and dried at 60 °C. The dried gel was subsequently carbonized at 1000 or 1600 °C for 2 h in a stream of argon gas (1 L min^−1^). The carbon samples derived from RF gels (RF carbons) were labeled with the carbonization temperature, namely RF-1000 and RF-1600.

### Characterization

Powder X-ray diffraction (XRD) measurements and Raman spectroscopy were employed to confirm that the samples had an amorphous carbon structure, using an X-ray diffractometer (X'Pert Pro, PANalytical, Netherlands, using Cu Kα radiation with a generator voltage of 45 kV and a tube current of 40 mA) and a Raman spectrometer (Jubin-Yvon iHR550, HORIBA, Japan, equipped with a Laser Quantum Ventus 532 nm laser using a power of 25 mW at an exposure time of 10 s), respectively. To investigate the morphology of the samples, scanning electron microscopy (SEM) measurements were performed on a scanning electron microscope (NEOSCOPE JCM-6000, JEOL, Japan, equipped with a tungsten filament at 15 kV).

Gas and vapor sorption analyses were performed to examine the surface area, pore size distribution, and pore volume of the HC samples by a gas/vapor adsorption measurement instrument (autosorb iQ, Quantachrome Instruments, USA). All samples were outgassed under vacuum at 350 °C for more than 4 h before measurement. Nitrogen (N_2_) and krypton (Kr) sorption measurements were carried out at 77.4 K (−196.75 °C). Unfortunately, the adsorption measurements of N_2_ on the HT samples could not reach equilibrium and it was therefore not possible to obtain reliable isotherms. This might be due to the large particle size and the abundant micropores in the samples as confirmed by carbon dioxide (CO_2_) sorption measurements. CO_2_ and water vapor (H_2_O) sorption were carried out at 273.15 K (0 °C) and 293.15 K (20 °C), respectively. From the results of Kr and CO_2_ adsorption, the specific surface area (SSA) was calculated using the Brunauer–Emmett–Teller (BET) method. The pore size distribution was obtained from CO_2_ adsorption measurements using the Monte Carlo method. The isotherms of H_2_O adsorption were used to estimate the pore size distribution using the equation reported by Wang et al. [34], which is based on the Mahle model [33]. Since our system could measure the adsorption amounts only up to *p*/*p*_0_ = 0.9, the measured value at this point was assumed to be the end point of the simulation curve at *p*/*p*_0_ = 1. The parameters were changed to get the simulation curves close to the actual adsorption isotherm curves. The TPVs were calculated from the last adsorption measurement point of CO_2_ (at *p*/*p*_0_ = 0.029) and H_2_O adsorption (at *p*/*p*_0_ = 0.9).

### Electrode preparation

The hard carbon electrodes were prepared by mixing HC powder (95 wt %) and polyvinylidene difluoride (PVdF, Kynar HSV 900, Arkema, France) (5 wt %) in *N*-methyl-2-pyrrolidinone (NMP, Sigma-Aldrich, Germany) for 10 min at 2000 rpm using a planetary centrifugal mixer (ARV-310, Thinky, Japan), such that the resulting slurry had a solid content of 0.8 mg mL^−1^. The viscous slurry was coated on a copper foil current collector with a thickness of 10 μm (MTI corporation, USA) on an automatic table-top coating machine (Coatema, Germany) using the doctor blade method, resulting in a wet film thickness of 90 µm. The HC loading of the electrodes was 3 ± 0.1 mg cm^−2^. After drying the films in an oven at 50 °C for 3 h, electrodes with a diameter of 10.95 mm were punched out. The electrodes were then dried in a glass oven (Büchi, Switzerland) under dynamic vacuum at 120 °C overnight and transferred into an argon-filled glovebox (H_2_O and O_2_ content <0.1 ppm, MBraun, Germany) without exposure to ambient air.

### Electrochemical characterization

In order to investigate the Li-ion capacity of the hard carbon active materials, three-electrode Swagelok T-cells were assembled in an argon-filled glovebox using four glass fiber separators (glass microfiber filter, 691, VWR, Germany, with a diameter of 11 mm), a lithium counter electrode (450 µm thick Li foil, Rockwood Lithium) and a Li reference electrode. The cells were then filled with 120 µL of electrolyte (1 M LiPF_6_ in EC/EMC = 3:7 (v/v), <20 ppm H_2_O, BASF, Germany). All electrochemical tests were carried out in a climatic chamber (Binder, Germany) at 25 °C using a battery cycler (Series 400, Maccor, USA). After connecting the cells to the potentiostat, the cells were rested for 2 h in order to assure proper wetting of the electrodes. Three charge-discharge cycles were performed at a rate of C/10 (35.5 mA g^−1^). The lower cut-off potential was 10 mV and the upper cut-off potential 1.5 V vs Li^+^/Li whereby the potential was controlled vs a Li reference electrode. Afterwards, the cells were cycled for 20 times with a rate of 1C (355 mA g^−1^). The Na-ion capacity of the hard carbon samples was also investigated in a three-electrode Swagelok T-cell using sodium metal (99.95%, Sigma-Aldrich, Germany) as counter and reference electrodes and 1 M NaPF_6_ (99%, Sigma-Aldrich, Germany) in EC/DEC = 1:1 (v/v) (<20 ppm H_2_O, BASF, Germany) as electrolyte. The same cut-off potentials and cycling procedure as for the Li-ion cells were used.

## Supporting Information

File 1 Additional figures.
