# Supplementary material for: Gas sorption porosimetry for the evaluation of hard carbons as anodes for Li- and Na-ion batteries
Source: Beilstein J Nanotechnol. 2020 Aug 14;11:1217–29. doi: 10.3762/bjnano.11.106 (PMC7431754; doi:10.3762/bjnano.11.106)
Supplement: File 1 — Additional figures. [file Beilstein_J_Nanotechnol-11-1217-s001.pdf]

BEILSTEIN JOURNAL OF NANOTECHNOLOGY

Supporting Information

for

Gas sorption porosimetry for the evaluation of hard carbons as anodes for Li- and Na-ion batteries

Yuko Matsukawa, Fabian Linsenmann, Maximilian A. Plass, George Hasegawa, Katsuro Hayashi and Tim-Patrick Fellinger

Beilstein J. Nanotechnol. **2020,** *11,* 1217–1229. doi:10.3762/bjnano.11.106

Additional figures

License and Terms: This is a supporting information file under the terms of the Creative Commons Attribution License (http://creativecommons.org/licenses/by/4.0). Please note that the reuse, redistribution and reproduction in particular requires that the authors and source are credited.
The license is subject to the *Beilstein Journal of Nanotechnology* terms and conditions: (https://www.beilstein-journals.org/bjnano)

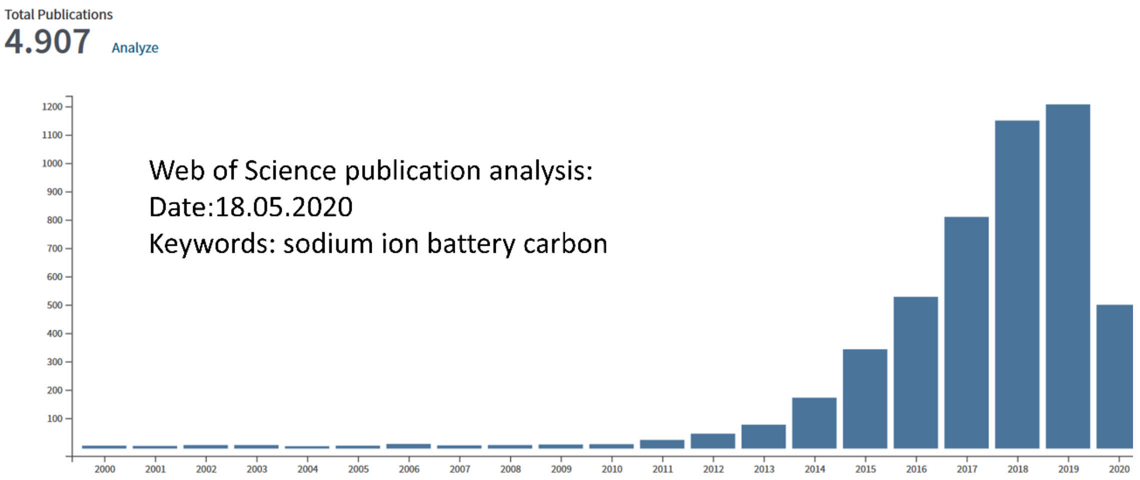

Figure S1: Publication trend since the year 2000 on carbons for SIBs according to Web of Science.

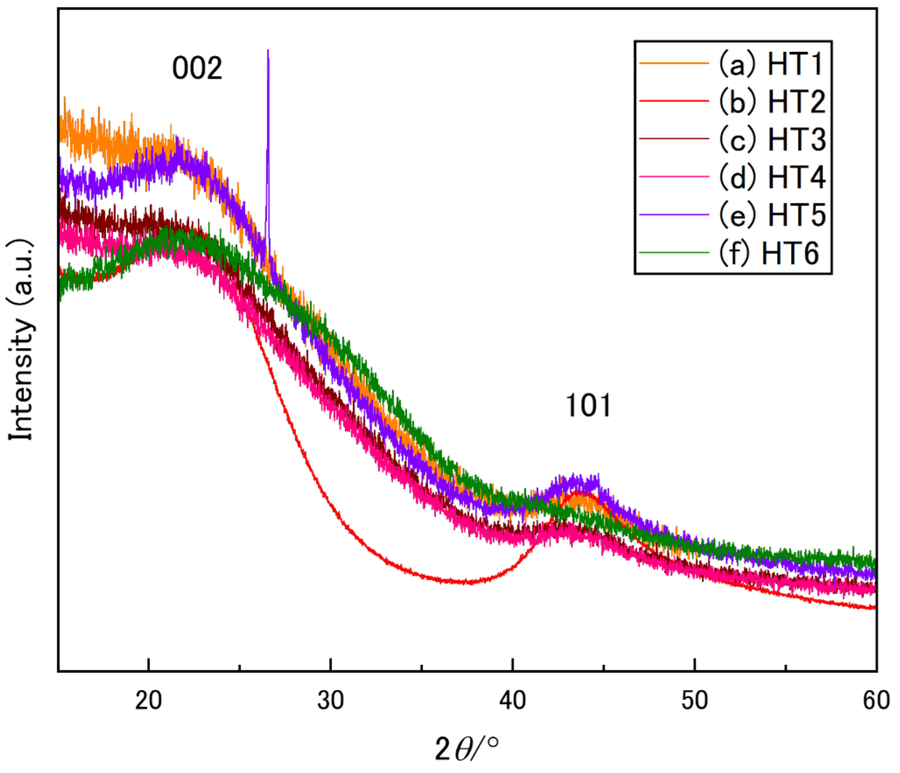

Figure S2: Powder X-ray diffraction pattern of HT2. d_{002} = 4.02 Å (2θ = 22.1°), d_{101} = 2.06 Å (2θ = 43.9°).

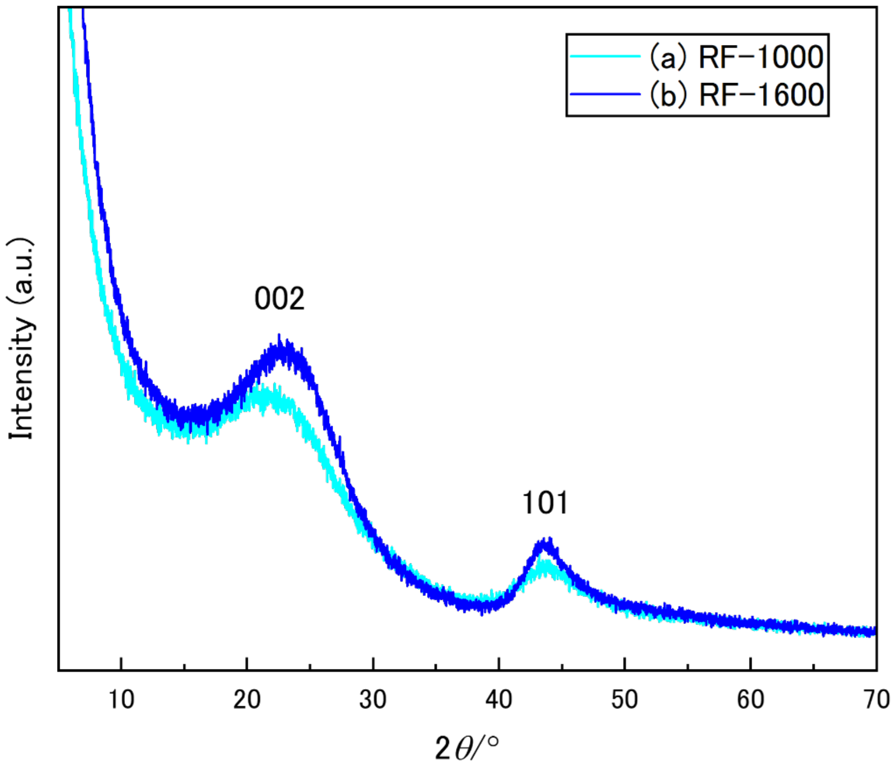

Figure S3: Powder X-ray diffraction patterns of (a) RF-1000 and (b) RF-1600. (a) d_{002} = 4.28 Å (2θ = 20.8°), d_{101} = 2.09 Å (2θ = 43.2°), (b) d_{002} = 3.94 Å (2θ = 22.5°), d_{101} = 2.06 Å (2θ = 44.0°).

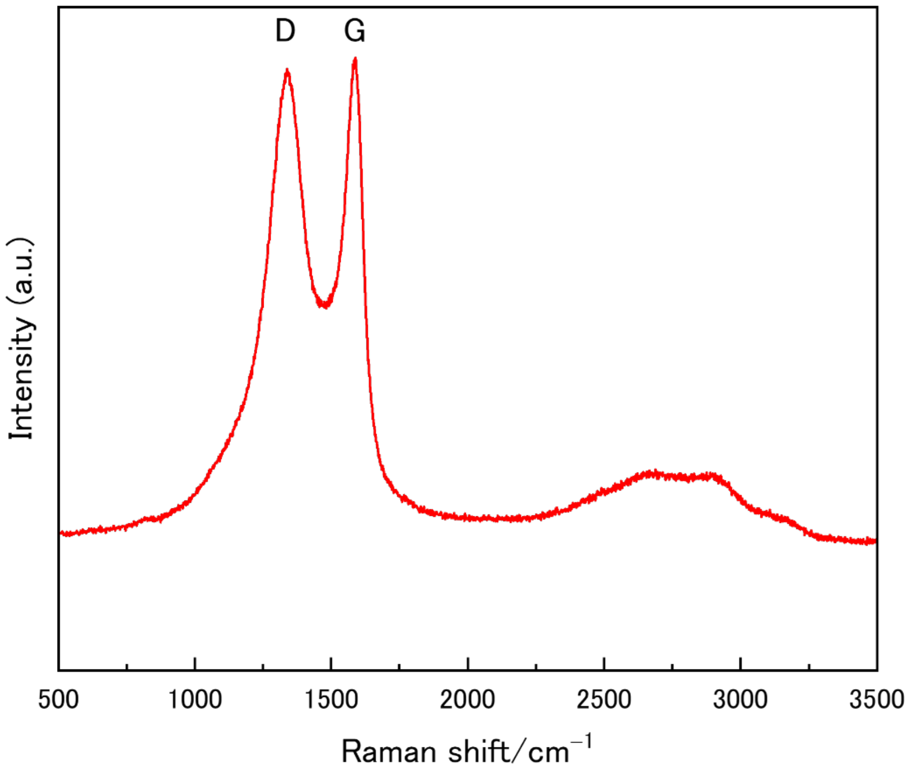

Figure S4: Raman spectrum of HT2. I_D/I_G = 0.98.

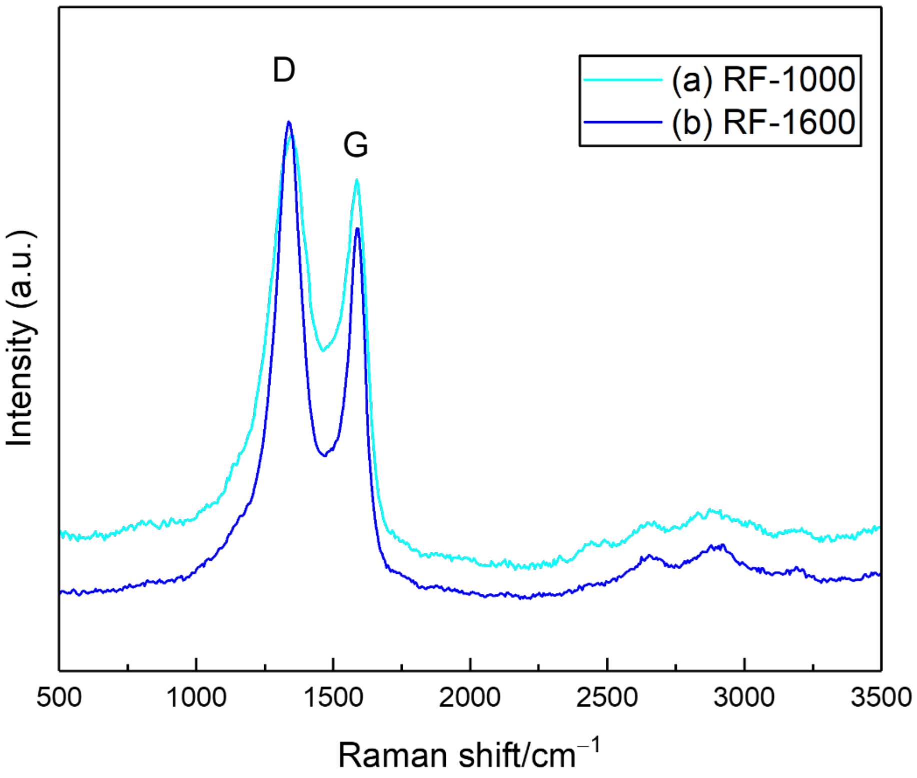

Figure S5: Raman spectra of (a) RF-1000 and (b) RF-1600. (a) I_D/I_G = 1.12, (b) I_D/I_G = 1.29. c) Micropore size distributions of the carbons calcined at different temperatures obtained from the corresponding N_2 isotherms by the Horvath–Kawazoe (HK) method. The carbons treated above 1600 °C possessed negligible micropores.

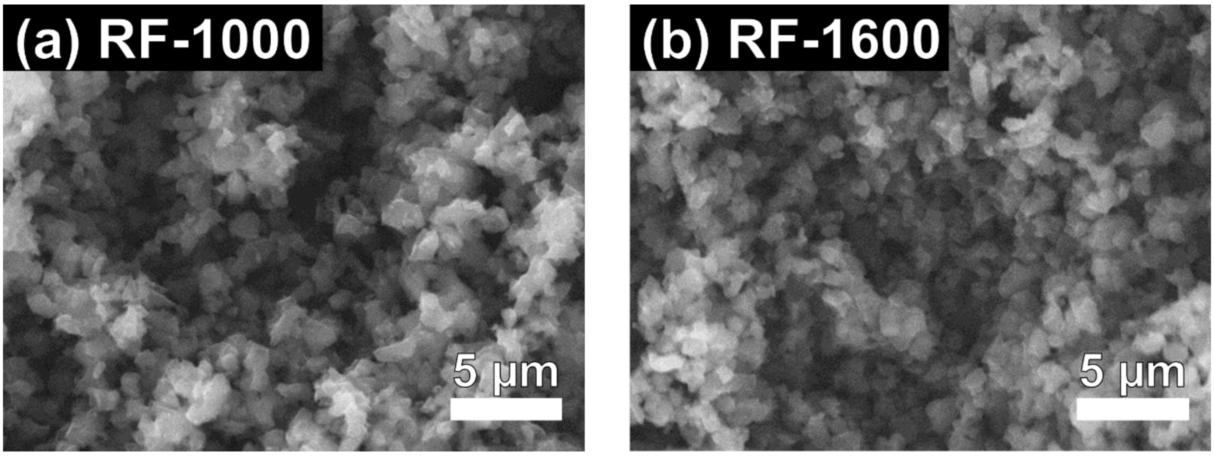

Figure S6: SEM images of RF carbons. (a) RF-1000, (b) RF-1600.

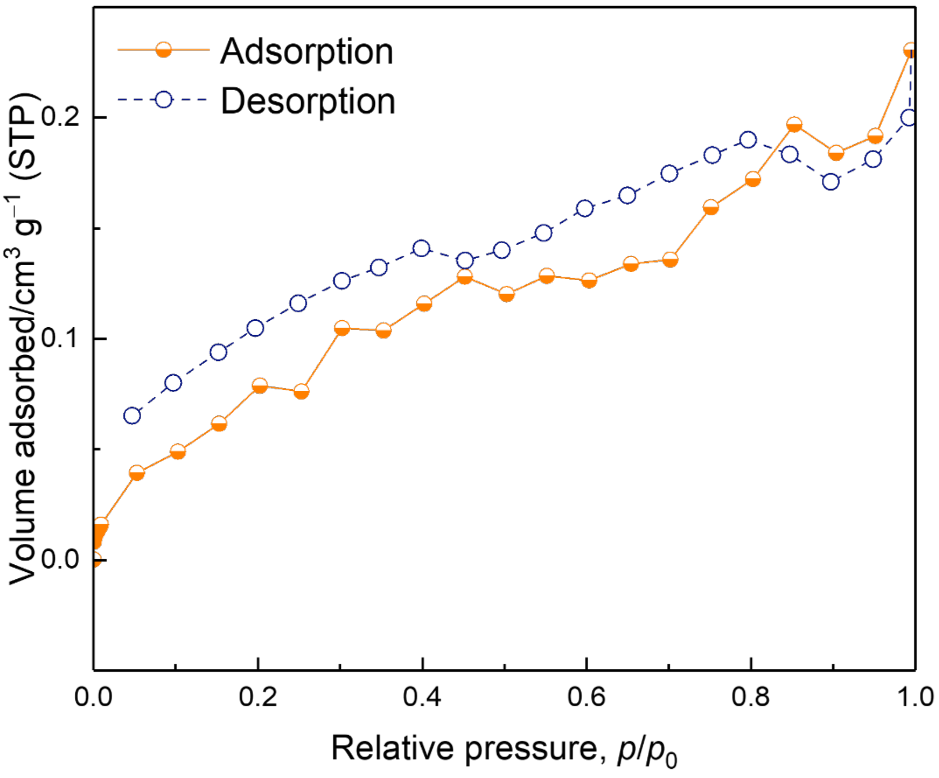

Figure S7: Failed N_2 sorption isotherms of HT1.

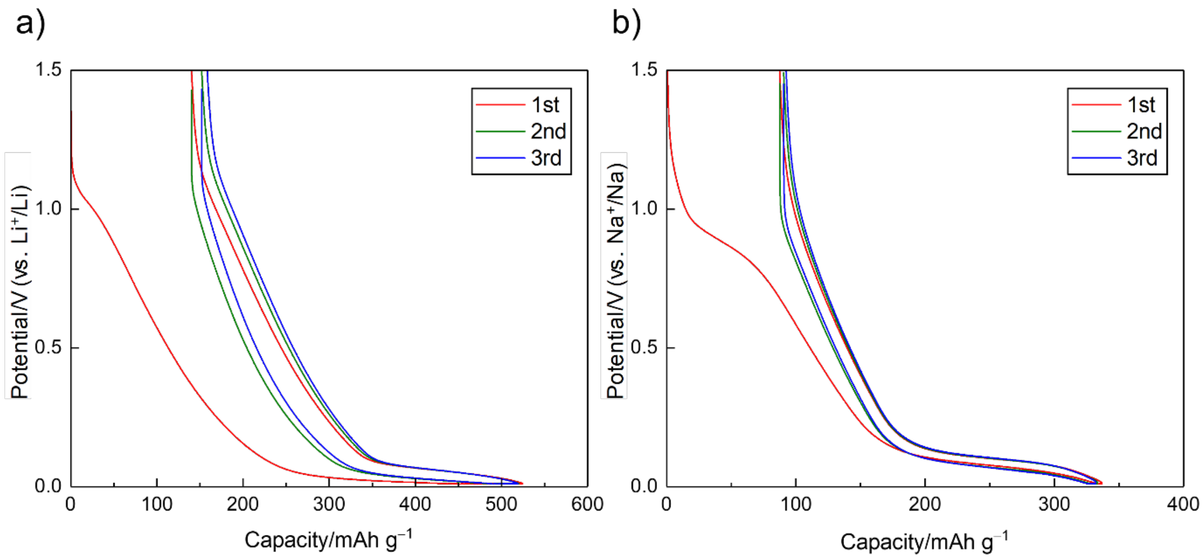

Figure S8: Charge-discharge curves of HT2 vs (a) Li and (b) Na metal electrode.
